# Supplementary material for: Modelling of the arteriovenous malformation embolization optimal scenario
Source: R Soc Open Sci. 2020 Jul 22;7(7):191992. doi: 10.1098/rsos.191992 (PMC7428277; doi:10.1098/rsos.191992)
Supplement: CABARET scheme [file rsos191992supp1.pdf]

A. A. Cherevko, T. S. Gologush, I. A. Petrenko, V. V. Ostapenko, V. A. Panarin, Modeling of the arteriovenous malformation embolization optimal scenario, Royal Society Open Science

CABARET scheme

Cauchy problem for Buckley-Leverett equation Let's set this scheme on a uniform rectangular grid

$$x_j = jh, \quad t_n = n\tau, \quad n \geq 0, \tag{1}$$

with constant steps h in space and τ in time. The time step is chosen from the stability condition

$$\tau = rh/B, \quad B = \max_{S\in[0,1]} f'(S), \tag{2}$$

where $r \in (0,1)$ is the Courant number. The CABARET scheme uses the flux variables $u_j^n = S(x_j, t_n)$ and conservative variables $U_{j+1/2}^n = S(x_{j+1/2}, t_n)$ defined respectively in integer x_j and half-integer $x_{j+1/2} = x_j + h/2$ spatial nodes of the difference grid.

First, consider the CABARET scheme application for numerical solution of the Cauchy problem for Buckley-Leverett equation with initial data

$$S(x,0) = S_0(x), \tag{3}$$

where $S_0(x)$ is the given piecewise continuous function. Let u_j^n, $U_{j+1/2}^n$ be the known numerical solution of Cauchy problem on the time layer t_n, with the following grid approximation of the initial function (3) for $n = 0$:

$$U_{j+1/2}^0 = S_0(x_{j+1/2}), \quad u_j^0 = \frac{U_{j-1/2}^0 + U_{j+1/2}^0}{2}\ . \tag{4}$$

Numerical solution u_j^{n+1}, $U_{j+1/2}^{n+1}$ on the next time layer t_{n+1} will be found using the CABARET scheme in four stages.

In the first stage, by difference equations

$$\frac{U_{j+1/2}^{n+1/2} - U_{j+1/2}^n}{\tau/2} + \frac{f_{j+1}^n - f_j^n}{h} = 0, \quad f_k^n = f(u_k^n), \tag{5}$$

the conservative variables $U_{j+1/2}^{n+1/2} = U(x_{j+1/2}, t_{n+1/2})$ are calculated on a half-integer time layer $t_{n+1/2} = t_n + \tau/2$. At the second stage, the numerical fluxes $f_j^{n+1/2} = f(u_j^{n+1/2})$ are found on the time layer $t_{n+1/2}$. At the third stage, by difference equations

$$\frac{U_{j+1/2}^{n+1} - U_{j+1/2}^n}{\tau} + \frac{f_{j+1}^{n+1/2} - f_j^{n+1/2}}{h} = 0 \tag{6}$$

the conservative variables $U_{j+1/2}^{n+1}$ are calculated on time layer t_{n+1}. At the fourth stage, the flux variables u_j^{n+1} are found on the time layer t_{n+1}. A description of the numerical algorithm in the second and fourth stages is given below.

Flux calculation and correction At the second stage, given that $f'(S) \geq 0$, preliminary flux values on the time layer $t_{n+1/2}$ are calculated in the following way

$$\bar{f}_j^{n+1/2} = f\left(\bar{u}_j^{n+1/2}\right), \ \bar{u}_j^{n+1/2} = \left(u_j^n + \bar{u}_j^{n+1}\right)/2, \ \bar{u}_j^{n+1} = 2U_{j-1/2}^{n+1/2} - u_{j-1}^n. \quad (7)$$

With standard double limiter

$$F(u, m, M) = \begin{cases} u, & m \leq u \leq M, \\ m, & u < m, \\ M, & u > M \end{cases} \quad (8)$$

these fluxes are corrected by the formula

$$\widetilde{f}_j^{n+1/2} = F\left(\overline{f}_j^{n+1/2}, m_j^n, M_j^n\right), \quad (9)$$

where $m_j^n = \min\left(f_{j-1/2}^n, f_j^n\right)$, $M_j^n = \max\left(f_{j-1/2}^n, f_j^n\right)$, $f_{j-1/2}^n = f(U_{j-1/2}^n)$. The correction (9) connected with the difference analogue of the maximum principle, which is satisfied by the exact solutions of the considered problem. However, such a correction does not provide [1] the performance for the difference scheme Harten TVD property [2] and therefore the monotonicity property in the Godunov sense [3].

Therefore, if on the n-th time layer in the neighborhood of the grid node $x_{k+1/2}$ the difference solution is locally monotonic, then an additional correction of the numerical fluxes is performed:

$$U_{k-3/2}^n \leq u_{k-1}^n \leq U_{k-1/2}^n \leq u_k^n \quad \Rightarrow \quad f_k^{n+1/2} = F_1\left(\widetilde{f}_k^{n+1/2}, \varphi_{k-1}^n\right), \quad (10)$$

$$U_{k-3/2}^n \geq u_{k-1}^n \geq U_{k-1/2}^n \geq u_k^n \quad \Rightarrow \quad f_k^{n+1/2} = F_2\left(\widetilde{f}_k^{n+1/2}, \varphi_{k-1}^n\right), \quad (11)$$

where

$$F_1(u, M) = \begin{cases} u, & u \leq M, \\ M, & u \geq M, \end{cases} \quad F_2(u, m) = \begin{cases} u, & u \geq m, \\ m, & u \leq m, \end{cases}$$
$$\varphi_{k-1}^n = \frac{\tau}{h}\left(U_{k-1/2}^n - u_{k-1}^n\right) + f_{k-1}^n. \quad (12)$$

This additional correction maintains the monotonicity of the difference solution $\left\{u_j^n, U_{j+1/2}^n\right\}$ with respect to the sequence of conservative variables $\left\{U_{j+1/2}^{n+1}\right\}$ on the time layer t_{n+1}. The proof of this result, formulated in [4], is carried out on the basis of a method outlined in [5] for the case of approximation by the CABARET scheme of a scalar conservation law with a convex flux. Note that the correction (10)–(12) does not reduce the accuracy of the scheme on local extremes located in the smooth parts of the calculated weak solution.

At the beginning of the fourth stage by the formulas

$$\widetilde{u}_j^{n+1} = 2u_j^{n+1/2} - u_j^n, \quad u_j^{n+1/2} = f^{-1}(f_j^{n+1/2}), \quad (13)$$

where f^{-1} is a function inverse to function f, the second preliminary values of the flux variables are determined on the time layer t_{n+1}. As shown in [6], the preservation in the CABARET scheme of monotonicity with respect to the sequence of conservative variables $\left\{U_{j+1/2}^{n+1}\right\}$ in the general case does not guarantee the monotonicity of the difference solution with respect to sequences $\left\{\widetilde{u}_j^{n+1}, U_{j+1/2}^{n+1}\right\}$ alternating values of flux $\widetilde{u}_j^{n+1}$ and conservative $U_{j+1/2}^{n+1}$ variables. Therefore, in conclusion, it is necessary to carry out an additional correction of flux variables

$$u_j^{n+1} = F\left(\widetilde{u}_j^{n+1}, m_j^{n+1}, M_j^{n+1}\right), \tag{14}$$

where $m_j^{n+1} = \min\left(U_{j-1/2}^{n+1}, U_{j+1/2}^{n+1}\right)$ and $M_j^{n+1} = \max\left(U_{j-1/2}^{n+1}, U_{j+1/2}^{n+1}\right)$, ensuring the monotony of the entire difference solution $\{u_j^{n+1}, U_{j+1/2}^{n+1}\}$ on the time layer t_{n+1}. Note that the last flux correction (14), which is carried out after applying the main divergent formula (6), does not violate of the CABARET scheme conservativity, since this correction can be considered as the first step in the flux calculating on the time layer t_{n+2}.

Initial-boundary value problem A significant advantage of the CABARET scheme compared to WENO type schemes is that this scheme is defined on a compact three-point spatial stencil located inside a single cell of the difference grid, and therefore, when calculating initial boundary-value problems, it is not necessary for the CABARET scheme to apply auxiliary asymmetric difference equations in the near-boundary grid nodes. To solve the Buckley-Leverett equation with the following initial and boundary conditions:

$$S(0, x) = S_0(x) \equiv 1, \quad x \in [0, L], \tag{15}$$

$$S(t, 0) = g(t) \in [0, 1], \quad t \in (0, T], \tag{16}$$

we divide the AVM segment $[0, L]$ into N equal parts of length $h = L/N$ and use the exact grid approximation

$$u_0^n = g(t_n) = g(n\tau) \tag{17}$$

of the boundary condition (16). The basic difference equations (5) and (6) approximating the conservation law $S_t + f(S)_x = 0$ are applied in all half-integer nodes $x_{j+1/2}$ of the difference grid with $j = \overline{0, N-1}$. The calculation of fluxes (7) and their correction (9)–(11), as well as the calculation of the flux functions (13), are carried out at all positive integer nodes x_j with $j = \overline{1, N}$. The correction (14) of flux functions is performed in all internal integer nodes x_j with $j = \overline{1, N-1}$.

References

[1] N. A. Zyuzina and V. V. Ostapenko. On the monotonicity of the CABARET scheme approximating a scalar conservation law with a convex flux. *Doklady Mathematics*, 93(1):69–73, jan 2016. doi: 10.1134/s1064562416010282.

[2] Ami Harten. High resolution schemes for hyperbolic conservation laws. *J. Comput. Phys.*, 49(3):357–393, mar 1983. doi: 10.1016/0021-9991(83)90136-5.

[3] S. K. Godunov. A difference scheme for numerical computation of discontinuous solution of hyperbolic equations. *Math. Sb.*, 47(3):271–306, 1959.

[4] V. V. Ostapenko and A. A. Cherevko. Application of the CABARET scheme for calculation of discontinuous solutions of the scalar conservation law with nonconvex flux. *Dokl. Phys.*, 62(10):470–474, oct 2017. doi: 10.1134/s1028335817100056.

[5] N. A. Zyuzina, O. A. Kovyrkina, and V. V. Ostapenko. On the monotonicity of the CABARET scheme approximating a scalar conservation law with an alternating characteristic field and convex flux function. *Math. Models Comput. Simul.*, 11(1):46–60, jan 2019. doi: 10.1134/s2070048219010186.

[6] O. A. Kovyrkina and V. V. Ostapenko. On monotonicity of two-layer in time cabaret scheme. *Math. Models Comput. Simul.*, 5(2):180–189, mar 2013. doi: 10.1134/s2070048213020051.
