# Supplementary material for: Modelling of the arteriovenous malformation embolization optimal scenario
Source: R Soc Open Sci. 2020 Jul 22;7(7):191992. doi: 10.1098/rsos.191992 (PMC7428277; doi:10.1098/rsos.191992)
Supplement: Clinical data [file rsos191992supp2.pdf]

A. A. Cherevko, T. S. Gologush, I. A. Petrenko, V. V. Ostapenko, V. A. Panarin, Modeling of the arteriovenous malformation embolization optimal scenario, Royal Society Open Science

Dependency table of the blood velocity v_b and blood pressure p in the artery on the volume average embolic agent concentration $1-\bar{S}$						
	Measurement number					
**Patient K**	1	2	3	4	5	6
$1-\bar{S}$	0,0000	0,2080	0,6053	0,7620	0,8700	1,0000
v_b [m/s]	0,2076	0,1625	0,0424	0,0562	0,0238	0,0000
p [mmHg]	58,26	66,33	80,71	78,53	76,53	68,68
**Patient T**	1	2	3	4	5	6
$1-\bar{S}$	0,0000	0,3240	0,5610	0,6120	0,8074	1,0000
v_b [m/s]	0,6254	0,4325	0,4304	0,4381	0,3869	0,0000
p [mmHg]	40,14	44,75	45,16	48,89	59,23	66,47
**Patient P**	1	2	3	4	5	
$1-\bar{S}$	0,0000	0,2500	0,5000	0,6250	1,0000	
v_b [m/s]	0,3875	0,0481	0,0125	0,0107	0,0000	
p [mmHg]	49,47	50,11	52,60	52,12	53,16	

Hemodynamic and geometric parameters of patients needed to calculate absolute permeability *K*							
Patient	*L* [10^(-2)*m]	*A* [10^(-4)*m^2]	*ω* [10^(-6)*m^2]	v_{b0} [m/s]	p_0 [mmHg]		K [m^2]
				Artery	Artery	Vein	
**K**	2,40	4,52160	4,52376	0,20760	58,26	40,00	8,19E-11
**T**	2,20	2,00000	4,52376	0,62540	40,14	40,00	6,67E-08
**P**	3,00	3,14150	3,14150	0,38750	49,47	40,00	3,68E-10
**S**	3,00	2,83520	2,54462	0,64490	65,70	40,00	2,03E-10
**I**	1,86	0,95030	4,90859	0,57240	46,48	12,32	4,83E-10
**B**	3,00	6,15734	5,30914	0,03282	61,20	21,01	6,34E-12
**A**	1,70	5,72538	4,90859	0,40779	32,60	25,00	2,35E-10
**C**	3,00	2,01056	1,32728	0,66527	33,44	27,08	6,21E-10
**Sh**	4,40	5,30914	1,53934	0,52415	24,83	6,27	1,08E-10
**M**	2,83	3,94070	1,32728	0,39375	69,16	60,56	1,31E-10
